# Níveis de Atividade Física e Qualidade de Vida em Pacientes Ambulatoriais com Arritmias Cardíacas e Função Ventricular Esquerda Preservada: Compreendendo Seu Perfil

**DOI:** 10.36660/abc.20250831

**Published:** 2026-06-19

**Authors:** Savia Christina Pereira Bueno, Patrícia Alves Oliveira, Márya Pagotti, Luciana Sacilotto, Gabrielle D’Arezzo Pessente, Pedro Gabriel Senger Braga, Aline L. Souza, Alexandra Regia Dantas Brigido, Carlos E. Negrão, Francisco C. C. Darrieux, Mauricio Scanavacca

**Affiliations:** 1 Universidade de São Paulo Instituto do Coração do Hospital das Clínicas Faculdade de Medicina São Paulo SP Brasil Instituto do Coração do Hospital das Clínicas da Faculdade de Medicina da Universidade de São Paulo, São Paulo, SP – Brasil; 2 Hospital Israelita Albert Einstein São Paulo SP Brasil Hospital Israelita Albert Einstein, São Paulo, SP – Brasil

**Keywords:** Arritmias Cardíacas, Exercício Físico, Qualidade de Vida

## Abstract

As arritmias cardíacas são condições heterogêneas, que podem limitar a atividade física (AF) e prejudicar a qualidade de vida (QV).

Descrever os níveis de AF e as barreiras percebidas, bem como avaliar suas associações com o fenótipo de arritmia e a QV em pacientes ambulatoriais de nível terciário com função do ventrículo esquerdo (VE) preservada, se faz necessário.

Este é um estudo piloto, transversal, que incluiu adultos com arritmias documentadas e fração de ejeção do VE (FEVE) ≥ 50%. A AF foi avaliada por meio da versão curta do *International Physical Activity Questionnaire* (IPAQ), e a QV pelo *12-Item Short Form Survey* (SF-12). As arritmias foram classificadas como taquicardia supraventricular (TSV), fibrilação atrial (FA) e/ou taquicardia atrial (TA) (incluindo flutter atrial quando aplicável), ventricular, hereditária ou múltipla. As associações foram analisadas por meio de testes do qui-quadrado, regressão multinomial e modelos de regressão linear.

Entre 202 participantes (idade média 50,5 ± 15,3 anos; 58,9% homens), 20,3% eram sedentários e 45,6% eram ativos ou muito ativos. A prevalência de comportamento sedentário foi maior entre pacientes com arritmias ventriculares (25,9%), arritmias hereditárias (35,1%) e arritmias múltiplas (25,0%) em comparação com aqueles com TSV (3,8%) e FA/TA (8,0%) (p = 0,043). Nos modelos ajustados, níveis de AF ativos ou muito ativos mostraram uma tendência a escores mais elevados no componente físico do SF-12 (p = 0,08), enquanto os escores do componente mental foram principalmente influenciados pelo *status* dos sintomas e pelo sexo.

Nesta coorte ambulatorial terciária com arritmias e FEVE preservada, a inatividade física concentrou-se em fenótipos de maior risco e esteve frequentemente associada a barreiras potencialmente modificáveis, incluindo orientação médica e falta de tempo. Níveis mais elevados de AF tenderam a estar associados a melhor estado de saúde física, sustentando a necessidade de aconselhamento individualizado baseado no risco e de estratégias supervisionadas para promover de forma segura a AF em pacientes com arritmias.

## Introdução

As arritmias englobam um amplo espectro de distúrbios do ritmo cardíaco associados ao aumento da morbidade e a um risco substancialmente elevado de morte súbita cardíaca. Além de sua complexidade eletrofisiológica, essas condições frequentemente geram incerteza entre pacientes e clínicos quanto à segurança da prática de atividade física (AF). Alguns indivíduos podem se exercitar sem preparo adequado ou supervisão médica, enquanto outros evitam completamente a AF devido ao receio de desencadear eventos arrítmicos. Ambas as situações podem comprometer os desfechos de saúde e a qualidade de vida (QV).^1-4^

As recomendações internacionais para adultos geralmente preconizam 150-300 minutos por semana de atividade aeróbica de intensidade moderada (ou 75-150 minutos por semana de atividade vigorosa), juntamente com atividades de fortalecimento muscular em pelo menos 2 dias por semana. Apesar do reconhecimento amplo de que a AF regular é benéfica e, em geral, segura para a maioria dos pacientes cardíacos, e da disponibilidade de orientações baseadas em evidências sobre prescrição e supervisão de exercícios, sua implementação na prática real permanece limitada. Essa lacuna é frequentemente impulsionada pela persistente incerteza tanto entre clínicos quanto entre pacientes.^1-4^

Diversos fatores podem contribuir para esse cenário, incluindo desafios na estratificação de risco e na prescrição individualizada de exercícios, familiaridade limitada com considerações específicas das arritmias e reconhecimento subótimo da heterogeneidade e das necessidades clínicas de pacientes com arritmias complexas ou hereditárias. Essas limitações podem, por sua vez, contribuir para níveis mais baixos de AF em subgrupos de maior risco, particularmente quando o aconselhamento é excessivamente restritivo, inconsistente ou não adaptado ao fenótipo arrítmico do indivíduo e aos gatilhos percebidos. Nesse contexto, orientações excessivamente cautelosas e comportamentos de evitação por medo podem, inadvertidamente, promover padrões sedentários, mesmo entre pacientes com FEVE preservada.^1-4^

A identificação de subgrupos de pacientes com maior risco de inatividade física ou de práticas de exercício inseguras é, portanto, essencial para orientar recomendações individualizadas e promover o engajamento seguro em AF. Assim, este estudo teve como objetivo descrever os níveis de AF e as barreiras percebidas, bem como explorar suas associações com o fenótipo de arritmia e a QV em uma coorte ambulatorial terciária com arritmias documentadas e FEVE preservada, incluindo síndromes arrítmicas hereditárias. Ao caracterizar os perfis de atividade e os fatores clínicos associados, este estudo busca identificar grupos de pacientes que possam se beneficiar de aconselhamento aprimorado, orientação estruturada para exercícios ou intervenções direcionadas para otimizar a segurança e a participação em AF.

## Métodos

### Desenho e local do estudo

Este foi um estudo clínico transversal, prospectivo, conduzido com base em uma amostra de conveniência de pacientes consecutivos. Como estudo piloto baseado em viabilidade, não foi realizado cálculo formal de tamanho amostral a priori; o tamanho da amostra foi definido pela capacidade esperada de recrutamento durante o período do estudo. O recrutamento teve início após aprovação pelo Comitê de Ética em Pesquisa institucional e a assinatura dos termos de consentimento informado.

Com um tamanho amostral de n = 202, a precisão em torno de uma prevalência de 20% de inatividade física é de aproximadamente ± 5,5% (intervalo de confiança de 95%), o que é adequado para análises descritivas e comparações geradoras de hipóteses entre fenótipos de arritmia neste contexto piloto. No entanto, os intervalos de confiança tornam-se mais amplos em subgrupos fenotípicos menores.

### População do estudo

Os participantes elegíveis foram pacientes ambulatoriais adultos consecutivos com arritmias cardíacas documentadas, incluindo taquicardia supraventricular (TSV, incluindo taquicardia por reentrada nodal atrioventricular e taquicardia por reentrada atrioventricular), fibrilação atrial (FA) e/ou taquicardia atrial (TA) (incluindo flutter atrial quando aplicável), ectopia ventricular/taquicardia ventricular não sustentada/taquicardia ventricular, síndromes arrítmicas hereditárias (por exemplo, síndrome do QT longo [SQTL] e síndrome de Brugada), e/ou parada cardíaca prévia, acompanhados em um ambulatório terciário de arritmias. Todos os participantes apresentavam fração de ejeção do ventrículo esquerdo (FEVE ≥ 50%) preservada na ecocardiografia transtorácica.

Os diagnósticos foram estabelecidos por meio da revisão de prontuários clínicos e documentação de eletrocardiografia (ECG) de 12 derivações, monitorização Holter, teste ergométrico e/ou avaliação de dispositivos implantáveis. As síndromes arrítmicas hereditárias foram identificadas com base em critérios clínicos aceitos, incluindo características eletrocardiográficas específicas do fenótipo, perfis de gatilhos e história familiar, com testes provocativos confirmatórios e/ou genéticos quando disponíveis. Condições como a síndrome de Wolff-Parkinson-White e outros distúrbios cardíacos hereditários também foram diagnosticadas por meio de ECG, monitorização Holter ou teste ergométrico.

Os critérios de exclusão incluíram indivíduos acamados, aqueles com deambulação prejudicada ou limitações severas de mobilidade, doença avançada com expectativa de vida < 1 ano e participação em estudos intervencionistas concomitantes. Como o recrutamento foi baseado em viabilidade e consecutivo, o número de pacientes triados, mas não incluídos, não foi registrado prospectivamente.

#### International Physical Activity Questionnaire

O *International Physical Activity Questionnaire* (IPAQ) é um instrumento padronizado utilizado para avaliar os níveis de AF em adultos com base na frequência, duração e intensidade. Os participantes relataram as atividades realizadas nos 7 dias anteriores, categorizadas como vigorosas, moderadas ou caminhada.

Os participantes foram classificados de acordo com as categorias padrão do IPAQ: muito ativo, ativo, irregularmente ativo A, irregularmente ativo B e sedentário. As categorias irregularmente ativo A e B representam indivíduos que realizaram alguma AF, mas não atingiram os critérios de frequência e/ou duração necessários para serem classificados como ativos ou muito ativos. Portanto, foram tratadas como categorias intermediárias ("insuficientemente ativos"), em vez de serem agrupadas com os grupos ativo ou sedentário.

Para as análises inferenciais, as categorias muito ativo e ativo foram combinadas devido ao pequeno número de participantes muito ativos (n = 7). Os modelos multinomiais compararam quatro categorias (ativo/muito ativo, irregularmente ativo A, irregularmente ativo B e sedentário), utilizando sedentário como categoria de referência.^5^

### Avaliação de qualidade de vida

O *12-Item Short Form Survey* (SF-12) é um instrumento validado, autoaplicável, que avalia a QV relacionada à saúde por meio de dois escores resumidos: o Componente Físico (CF) e o Componente Mental (CM), ambos variando de 0 a 100, com escores mais elevados indicando melhor percepção de saúde.^6,7^

### Desfechos

Os desfechos primários foram: i) a prevalência de inatividade física avaliada pelo IPAQ; e ii) os escores do CF e do CM do SF-12. Os desfechos secundários incluíram barreiras autorrelatadas para a prática de exercícios e associações exploratórias entre o nível de AF e a QV.

### Análise estatística

As variáveis contínuas são apresentadas como média ± desvio padrão (DP) quando aproximadamente distribuídas normalmente, e como mediana (intervalo interquartil) caso contrário. As variáveis categóricas são apresentadas como contagens e porcentagens (n [%]). As suposições de distribuição foram avaliadas visualmente por meio de histogramas e gráficos Q-Q.

As comparações entre grupos para variáveis categóricas foram realizadas utilizando o teste do qui-quadrado de Pearson ou o teste exato de Fisher, conforme apropriado. A idade entre as categorias do IPAQ foi comparada por meio de análise de variância de uma via, com diagnósticos dos resíduos realizados para avaliar as suposições do modelo.

A associação entre a categoria do IPAQ (ativo/muito ativo, irregularmente ativo A, irregularmente ativo B, sedentário) e o grupo de arritmia foi avaliada por meio do teste do qui-quadrado de Pearson e posteriormente examinada por regressão logística multinomial (desfecho de referência: sedentário; grupo de arritmia de referência: hereditário), ajustada para idade.

As associações entre os escores do CF do SF-12 e a categoria do IPAQ foram avaliadas por meio de modelos de regressão linear multivariável ajustados para idade, sexo e *status* dos sintomas. Os modelos para os escores do CM foram ajustados apenas para sexo e *status* dos sintomas. Um valor de alfa bicaudal de 0,05 foi considerado estatisticamente significativo. Devido à natureza exploratória e geradora de hipóteses do estudo, não foi aplicado ajuste formal para múltiplas comparações.

## Resultados

### Características demográficas e clínicas da amostra

A população do estudo apresentou idade média de 50,5 ± 15,3 anos, e 119 participantes (58,9%) eram do sexo masculino. As características demográficas e clínicas basais estão resumidas na Tabela 1. A maioria dos participantes era assintomática (110 [54,4%]), e uma minoria utilizava terapia com dispositivos, incluindo cardioversores-desfibriladores implantáveis (CDIs) em 45 (22,3%) e marca-passos em 6 (3,0%).

**Tabela 1:** Características demográficas e clínicas basais

Variável		Valor
Tamanho da amostra, n		202
Idade, anos (média ± DP)		50,5 ± 15,3
Sexo masculino, n (%)		119 (58,9)
Hipertensão, n (%)		65 (32,2)
Diabetes melito, n (%)		34 (16,8)
*Status* assintomático, n (%)		110 (54,4)
Cardioversor-desfibrilador implantável, n (%)		45 (22,3)
Marca-passo, n (%)		6 (3,0)
Categoria de IMC, n (%)		
	Peso normal	62 (30,7)
	Sobrepeso	81 (40,1)
	Obesidade classe I	39 (19,3)
	Obesidade classe II	9 (4,5)
	Obesidade classe III	10 (4,9)
	Valores ausentes	1 (0,5)

DP: desvio padrão; IMC: índice de massa corporal

As arritmias foram categorizadas em cinco grupos: i) TSV, ii) arritmias ventriculares, iii) arritmias hereditárias, iv) arritmias múltiplas e v) FA e/ou TA. O flutter atrial poderia estar presente nesta coorte e é descrito entre as arritmias documentadas; no entanto, para as análises de subgrupos predefinidas, a categoria atrial foi denominada FA/TA para garantir consistência entre tabelas e modelos. A distribuição basal dos grupos de arritmia está apresentada na Tabela 2.

**Tabela 2:** Grupos de arritmia (classificação clínica pré-especificada)

Grupo de arritmia	n (%)
TSV	26 (12,9%)
FA/TA	50 (24,8%)
Arritmias ventriculares	81 (40,1%)
Arritmias hereditárias	37 (18,3%)
Arritmias múltiplas	8 (4,0%)

FA: fibrilação atrial; TA: taquicardia atrial; TSV: taquicardia supraventricular.

### Atividade física

**Figura 1:**
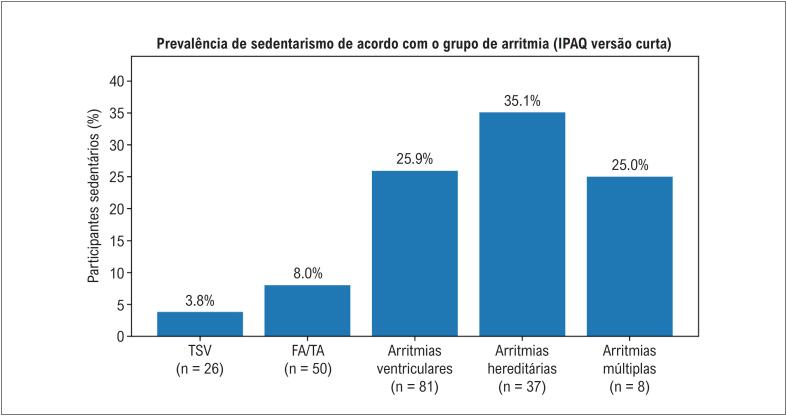
Proporção de participantes sedentários de acordo com o grupo de arritmia (IPAQ versão curta). FA: fibrilação atrial; IPAQ: International Physical Activity Questionnaire; TA: taquicardia atrial; TSV: taquicardia supraventricular.

As categorias gerais do IPAQ estão apresentadas na Tabela 3. No total, 41 participantes (20,3%) eram sedentários, 85 (42,1%) eram ativos e 7 (3,5%) eram muito ativos (ativo/muito ativo combinados: 92 [45,6%]). A prevalência de comportamento sedentário foi maior entre participantes com arritmias ventriculares (25,9%), arritmias hereditárias (35,1%) e arritmias múltiplas (25,0%), em comparação com aqueles com TSV (3,8%) e FA/TA (incluindo flutter atrial quando aplicável) (8,0%) (p = 0,043) (Figura 1; Tabela 4). Esses achados foram observados apesar de todos os participantes apresentarem FEVE preservada e de a coorte ser predominantemente assintomática (Tabela 1).

**Tabela 3:** Categorias de atividade física segundo o IPAQ (versão curta)

Categoria do IPAQ	n (%)
Muito ativo	7 (3,5%)
Ativo	85 (42,1%)
Irregularmente ativo A	35 (17,3%)
Irregularmente ativo B	34 (16,8%)
Sedentário	41 (20,3%)

IPAQ: International Physical Activity Questionnaire.

**Tabela 4:** Classificação do IPAQ por grupo de arritmia

Grupo de arritmia	Total, n	Muito ativo, n	Ativo, n	Irregularmente ativo A, n	Irregularmente ativo B, n	Sedentário, n (%)
TSV	26	4	9	7	5	1 (3,8)
FA/TA	50	3	26	10	7	4 (8,0)
Arritmias ventriculares	81	0	34	9	17	21 (25,9)
Arritmias hereditárias	37	0	13	8	3	13 (35,1)
Arritmias múltiplas	8	0	3	1	2	2 (25,0)

FA: fibrilação atrial; IPAQ: *International Physical Activity Questionnaire*; TA: taquicardia atrial; TSV: taquicardia supraventricular.

### Modelo multinomial para classificação do International Physical Activity Questionnaire

Na análise de regressão logística multinomial (categoria de referência: IPAQ sedentário; grupo de arritmia de referência: hereditário; ajustado para idade), os grupos TSV e FA/TA apresentaram maiores chances de serem classificados como ativos ou muito ativos em comparação com o grupo de arritmias hereditárias (Tabela 5).

### Qualidade de vida

A maioria dos participantes classificou sua saúde como boa (65%) e relatou mínima dificuldade nas atividades diárias. No entanto, 39% apresentaram menor desempenho físico e 43% relataram redução do desempenho mental. Dor e sofrimento emocional foram, em geral, leves ou ausentes. Homens e indivíduos assintomáticos apresentaram escores mais elevados de CF e CM.

Nos modelos ajustados, níveis de AF ativos ou muito ativos mostraram uma tendência a escores mais elevados de CF (Tabela 6), enquanto a categoria de AF não esteve independentemente associada aos escores de CM (Tabela 7).

### Achados adicionais

As principais razões relatadas para a inatividade física foram "outros" (41,5%), falta de tempo (24,4%) e orientação médica (19,5%). Apesar disso, a maioria dos participantes sedentários expressou desejo de praticar exercícios. Após o diagnóstico de arritmia, 66% dos participantes continuaram se exercitando e 85% mantiveram atividade sexual.

### Relação entre atividade física e qualidade de vida

Os participantes classificados como fisicamente ativos apresentaram escores mais elevados de CF em comparação com indivíduos inativos. Além disso, a ausência de sintomas esteve associada a melhores desfechos de QV.

## Discussão

Pesquisas demonstram consistentemente que níveis mais elevados de AF estão associados à melhora do bem-estar físico e psicológico. As recomendações atuais orientam 150-300 minutos por semana de atividade aeróbica de intensidade moderada (ou 75-150 minutos por semana de atividade vigorosa), juntamente com atividades de fortalecimento muscular em pelo menos 2 dias por semana. Em pacientes com arritmias, o aconselhamento para exercício deve considerar o substrato arrítmico, a carga de sintomas, os gatilhos e o uso de terapia com dispositivos. Exercícios mais vigorosos ou competitivos devem ser considerados dentro de uma abordagem individualizada, baseada na estratificação de risco, otimização do tratamento e tomada de decisão compartilhada.^1-4^

A evidência existente tem se concentrado predominantemente em coortes específicas por fenótipo ou em populações com dispositivos. Na FA, o estudo CHAMPLAIN-AF (n = 619) relatou uma mediana de 100 minutos/semana de AF moderada a vigorosa (AFMV) e um tempo sentado de 6 horas/dia. Notavelmente, 56% dos participantes não atingiam as metas recomendadas pelas diretrizes, 54% desconheciam ou tinham incerteza sobre as recomendações de AFMV, e 72% acreditavam que a AF deveria fazer parte do manejo da FA. É importante destacar que essa coorte não foi restrita a pacientes com FEVE preservada e incluiu indivíduos com insuficiência cardíaca (IC) ou cardiomiopatia. De forma semelhante, estudos com acelerômetros em pacientes com dispositivos (por exemplo, usuários de CDI ou submetidos à terapia de ressincronização cardíaca) demonstraram que níveis mais baixos de AF estão associados à hospitalização por IC e mortalidade; no entanto, essas coortes geralmente apresentam predominância de FEVE reduzida.

Nesse contexto, os dados especificamente voltados para pacientes ambulatoriais com arritmias e FEVE preservada permanecem limitados. Isso levanta a possibilidade de que o próprio diagnóstico, associado a orientações excessivamente restritivas ou não individualizadas, possa inadvertidamente promover comportamento sedentário. Poucos estudos avaliaram níveis de atividade e barreiras modificáveis entre diferentes fenótipos de arritmia dentro de uma única coorte ambulatorial terciária com FEVE preservada.^8,9^ A novidade do presente estudo não reside na direção das associações, mas na distribuição da inatividade estratificada por fenótipo e das barreiras modificáveis nesse contexto clínico específico.

Nesta coorte, a inatividade física foi frequente e concentrou-se em fenótipos clinicamente de maior risco, incluindo os grupos de arritmias ventriculares e hereditárias, embora esses achados devam ser interpretados com cautela devido ao menor tamanho dos subgrupos (Figura 1). A prevalência de sedentarismo foi mais elevada no grupo de arritmias hereditárias (35,1%) e permaneceu aumentada nos grupos de arritmias ventriculares (25,9%) e múltiplas (25,0%), em comparação com TSV (3,8%) e FA/TA (8,0%). Notavelmente, 45,6% dos participantes foram classificados como ativos ou muito ativos, e a maioria (54,4%) era assintomática, relatando boa saúde geral e mínimas limitações nas atividades diárias.

**Figura 2:**
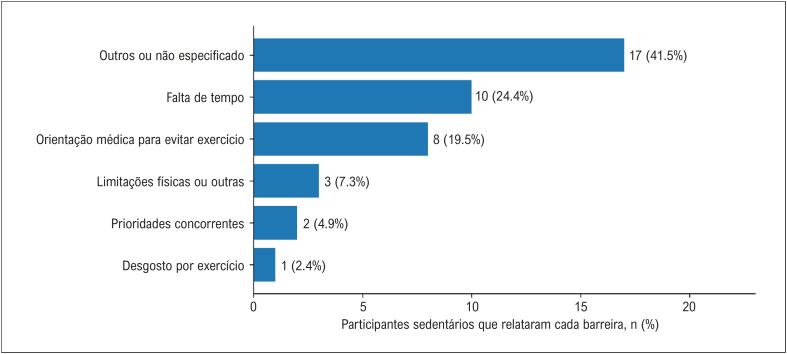
Principais barreiras relatadas para a atividade física entre participantes sedentários (n = 41). As barras representam o número e a porcentagem de participantes que relataram cada barreira. Notavelmente, 39 de 41 participantes (95,1%) expressaram disposição para se tornarem fisicamente ativos.

Esses achados sugerem que fatores além da carga de sintomas, como percepção de risco, práticas de aconselhamento, efeitos de medicamentos e restrições práticas, podem desempenhar papel relevante na limitação da AF entre fenótipos de maior risco. Entre os participantes sedentários, as barreiras mais frequentemente relatadas foram "outros", falta de tempo e orientação médica para evitar exercício. Apesar disso, a maioria expressou disposição para praticar AF (Figura 2).

Nos modelos multinomiais ajustados por idade, TSV e FA/TA estiveram associados a maiores chances de serem classificados como ativos ou muito ativos em comparação com arritmias hereditárias (Tabela 5). Isso sustenta um padrão específico por fenótipo, no qual substratos arrítmicos hereditários podem estar associados a maior restrição, percepção aumentada de risco ou comportamentos, que evitam, por medo mesmo em uma coorte predominantemente assintomática com FEVE preservada.

**Tabela 5:** Regressão logística multinomial: ativo/muito ativo vs sedentário (grupo de arritmia de referência: hereditário; ajustado para idade)

Preditor	OR	IC95%	Valor de p
Intercepto	—	—	0,459
Idade	1,01	0,99-1,04	0,308
TSV	12,06	1,36-106,90	0,025
FA/TA	5,77	1,52-21,96	0,010
Arritmias ventriculares	1,42	0,54-3,74	0,479
Arritmias múltiplas	1,27	0,18-9,10	0,811

IC95%: intervalo de confiança de 95%; FA: fibrilação atrial; OR: odds ratio; TA: taquicardia atrial; TSV: taquicardia supraventricular.

Além das diferenças por fenótipo, o nível de AF apresentou tendência de associação com a QV relacionada à saúde no domínio físico. Nos modelos lineares ajustados, indivíduos ativos ou muito ativos mostraram tendência a escores mais elevados de CF no SF-12 em comparação com participantes sedentários, enquanto as categorias irregularmente ativas não diferiram substancialmente dos sedentários (Tabela 6). Em contraste, os escores de CM do SF-12 não apresentaram associação clara com as categorias de atividade e pareceram ser mais influenciados pelo *status* dos sintomas e pelo sexo (Tabela 7).

**Tabela 6:** Regressão linear multivariável para o escore de CF do SF-12 de acordo com o nível de atividade física, ajustado para idade, sexo e status dos sintomas

Preditor	Coeficiente β	EP	Valor de p
Intercepto	47,38	2,55	< 0,001
Ativo/muito ativo	2,94	1,67	0,080
Irregularmente ativo A	0,23	2,08	0,910
Irregularmente ativo B	–0,04	2,04	0,982
Idade (por ano)	–0,13	0,04	0,001
Sexo masculino	1,70	1,29	0,189
*Status* assintomático	5,62	1,30	< 0,001

CF: Componente Físico; EP: erro padrão; SF-12: 12-Item Short Form Survey.

**Tabela 7:** Regressão linear multivariável para o escore de CM do SF-12 de acordo com o nível de atividade física, ajustado para sexo e status dos sintomas

Preditor	Coeficiente β	EP	Valor de p
Intercepto	42,83	1,82	< 0,001
Ativo/muito ativo	–0,69	1,81	0,703
Irregularmente ativo A	–0,69	2,23	0,757
Irregularmente ativo B	0,82	2,22	0,711
Sexo masculino	3,58	1,40	0,011
*Status* assintomático	3,48	1,40	0,014

CM: Componente Mental; EP: erro padrão; SF-12: 12-Item Short Form Survey.

Entre os participantes sedentários, as principais barreiras relatadas foram falta de tempo e orientação médica para evitar exercício; no entanto, a maioria expressou disposição para se tornar fisicamente ativa. Em conjunto, esses achados evidenciam uma lacuna prática entre as recomendações de exercício baseadas em diretrizes e o aconselhamento realizado na prática clínica em serviços terciários de arritmia.

O *status* basal de sintomas não esteve claramente associado às categorias do IPAQ, enquanto se associou de forma consistente aos domínios do SF-12. Isso sugere que o comportamento sedentário pode refletir não apenas a carga de sintomas, mas também fatores como percepção de risco, práticas de aconselhamento e medo de desencadear arritmias.

Apesar do amplo reconhecimento de que a AF regular é benéfica e em geral segura para a maioria dos pacientes cardíacos, sua implementação permanece limitada. Essa lacuna pode ser impulsionada por desafios na estratificação de risco e na prescrição individualizada de exercícios, familiaridade limitada com aspectos específicos das arritmias e reconhecimento incompleto da heterogeneidade e das necessidades clínicas de pacientes com arritmias complexas ou hereditárias.

Embora esta coorte não tenha sido desenhada para representar atletas ou praticantes de esportes competitivos, a cardiologia do esporte e diretrizes específicas por doença fornecem um arcabouço prático, baseado em fenótipo, para contextualizar o aconselhamento sobre exercício na prática clínica. Assim, considerações orientadas por diretrizes de acordo com a categoria de arritmia são apresentadas na Tabela 8; estas não devem ser interpretadas como critérios formais de elegibilidade para participação em esportes competitivos.

**Tabela 8:** Recomendações e considerações para exercício orientadas por diretrizes de acordo com a categoria de arritmia (resumo)

Categoria de arritmia	Recomendação geral (resumo)	Principais referências
TSV/pré-excitação (estável, controlada)	A atividade aeróbica recreativa de intensidade moderada é geralmente incentivada; avaliação individualizada é recomendada na presença de pré-excitação com características de alto risco.	Sharma et al., 2021^2^
FA/TA	A atividade física regular é incentivada; recomenda-se pelo menos 150 min/semana de atividade de intensidade moderada (ou equivalente), quando clinicamente apropriado. A tomada de decisão compartilhada deve considerar a carga de sintomas, o controle da frequência e as comorbidades.	Sharma et al., 2021^2^; Joglar et al., 2024^4^
Arritmias ventriculares/TV (FEVE preservada)	A prescrição de exercício deve ser individualizada. Exercícios intensos ou competitivos devem ser evitados em fenótipos de maior risco até adequada estratificação de risco e otimização do tratamento; programas supervisionados podem ser apropriados.	Sharma et al., 2021^2^; Zeppenfeld et al., 2022^3^
Síndromes arrítmicas hereditárias (p.ex., SQTL, síndrome de Brugada, TVPC)	As restrições ao exercício devem ser individualizadas de acordo com genótipo, fenótipo e perfil de gatilhos. Deve-se enfatizar o planejamento do exercício orientado por médico e estratégias de mitigação de risco.	Sharma et al., 2021^2^; Gil et al., 2025^10^; Chen et al., 2022^11^
Portadores de CDI	A participação em esportes competitivos ou vigorosos foi historicamente restrita; evidências contemporâneas sustentam a tomada de decisão individualizada. Programação do dispositivo, decisão compartilhada e avaliação de risco específica do esporte são essenciais.	Sharma et al., 2021^2^

CDI: cardioversor-desfibrilador implantável; FA: fibrilação atrial; FEVE: fração de ejeção do ventrículo esquerdo; SQTL: síndrome do QT longo; TA: taquicardia atrial; TSV: taquicardia supraventricular; TV: taquicardia ventricular; TVPC: TV polimórfica catecolaminérgica.

As síndromes arrítmicas hereditárias frequentemente ocorrem na ausência de doença cardíaca estrutural evidente. Evidências disponíveis sugerem altas taxas de comportamento sedentário em determinadas populações (por exemplo, coortes com síndrome de Brugada), sustentando a hipótese de que preocupações relacionadas ao diagnóstico, práticas de aconselhamento e comportamentos de evitação por medo podem contribuir para níveis mais baixos de AF, mesmo quando a função sistólica está preservada. Por outro lado, em SQTL, algumas coortes relatam níveis de atividade autorreferidos semelhantes aos de controles, porém com desempenho físico reduzido, evidenciando a possível influência do tratamento (por exemplo, betabloqueadores) e de limitações percebidas.^10,11^

A farmacoterapia também pode influenciar o comportamento e a tolerância ao exercício. Os betabloqueadores, terapia fundamental em diversos contextos de arritmia, podem atenuar a resposta cronotrópica e reduzir a capacidade percebida de exercício, potencialmente reforçando a inatividade.^12,13^ Isso ressalta a importância do aconselhamento individualizado para diferenciar efeitos farmacológicos esperados de verdadeira intolerância ao exercício, bem como o papel de programas supervisionados na promoção de atividade segura e progressiva, mantendo o controle das arritmias.^2-4^

De modo geral, esses achados apoiam a avaliação sistemática da AF e de suas barreiras por meio de instrumentos padronizados, seguida de aconselhamento baseado em risco. Também destacam a necessidade de vias assistenciais estruturadas, potencialmente integradas a programas de reabilitação cardíaca, para oferecer prescrição supervisionada de exercícios, especialmente em fenótipos de maior risco. A principal contribuição deste estudo é fornecer dados pragmáticos, de mundo real, provenientes de uma coorte ambulatorial terciária com arritmias e FEVE preservada, incluindo a distribuição da inatividade entre subgrupos clinicamente relevantes e a identificação de barreiras modificáveis.

### Limitações do estudo

A AF foi autorreferida por meio do IPAQ, o que pode estar sujeito a viés de memória, e o delineamento transversal impede inferência causal. Trata-se de uma coorte de centro único, de nível terciário, com FEVE preservada; portanto, os achados podem não ser generalizáveis para populações com arritmias na comunidade ou para pacientes com FEVE reduzida. Além disso, as estimativas em subgrupos foram imprecisas para fenótipos menores, particularmente o grupo de arritmias múltiplas. Não foram avaliados a participação em esportes competitivos, a AF mensurada objetivamente ou o detalhamento da dose e intensidade da farmacoterapia.

## Conclusão

Em uma coorte ambulatorial terciária de pacientes com arritmias e FEVE preservada, a inatividade física concentrou-se em fenótipos de maior risco e esteve frequentemente associada a barreiras potencialmente modificáveis, incluindo orientação médica e falta de tempo. Níveis mais elevados de AF tenderam a estar associados a melhor estado de saúde física, reforçando a necessidade de aconselhamento individualizado baseado em risco e de estratégias supervisionadas para promover de forma segura a AF em populações com arritmias.

## Data Availability

Os conteúdos subjacentes ao texto da pesquisa estão contidos no manuscrito.
