# Supplementary material for: Níveis de Atividade Física e Qualidade de Vida em Pacientes Ambulatoriais com Arritmias Cardíacas e Função Ventricular Esquerda Preservada: Compreendendo Seu Perfil
Source: Arq Bras Cardiol. 2026 Jun 19;123(5):e20250831. [Article in Portuguese] doi: 10.36660/abc.20250831 (PMC13399171; doi:10.36660/abc.20250831)
Supplement: Translation

# Translation: Physical Activity Levels and Quality of Life in Outpatients with Cardiac Arrhythmias and Preserved Left Ventricular Function: Understanding Their Profile

**DOI:** 10.36660/abc.20250831i

**Authors:** Savia Christina Pereira Bueno, Patrícia Alves Oliveira, Márya Pagotti, Luciana Sacilotto, Gabrielle D’Arezzo Pessente, Pedro Gabriel Senger Braga, Aline L. Souza, Alexandra Regia Dantas Brigido, Carlos E. Negrão, Francisco C. C. Darrieux, Mauricio Scanavacca

**Affiliations:** 1 ão Paulo SP Brazil Instituto do Coração do Hospital das Clínicas da Faculdade de Medicina da Universidade de São Paulo, São Paulo, SP – Brazil; 2 São Paulo SP Brazil Hospital Israelita Albert Einstein, São Paulo, SP – Brazil

**Keywords:** Cardiac Arrhythmias, Exercise, Quality of Life

## Abstract

Cardiac arrhythmias are heterogeneous conditions that may limit physical activity (PA) and impair quality of life (QoL).

To describe levels of PA and perceived barriers as well as to assess their associations with arrhythmia phenotype and QoL in tertiary care outpatients with preserved left ventricular (LV) function.

This pilot cross-sectional study enrolled adults with documented arrhythmias and LV ejection fraction (LVEF) ≥ 50%. PA was assessed using the International Physical Activity Questionnaire (IPAQ) short form, and QoL using the 12- Item Short Form Survey (SF-12). Arrhythmias were classified as supraventricular tachycardia (SVT), atrial fibrillation (AF) and/or atrial tachycardia (AT) (including atrial flutter when applicable), ventricular, inherited, or multiple. Associations were analyzed using chi-square tests, multinomial regression, and linear regression models.

Among 202 participants (mean age 50.5 ± 15.3 years; 58.9% men), 20.3% were sedentary and 45.6% were active or very active. The prevalence of sedentary behavior was higher among patients with ventricular arrhythmias (25.9%), inherited arrhythmias (35.1%), and multiple arrhythmias (25.0%) compared with those with SVT (3.8%) and AF/AT (8.0%) (p = 0.043). In adjusted models, active or very active PA showed a trend toward higher SF-12 physical component scores (p = 0.08), whereas mental component scores were primarily influenced by symptom status and sex.

In this tertiary outpatient cohort with arrhythmias and preserved LVEF, physical inactivity clustered among higher-risk phenotypes and was frequently associated with potentially modifiable barriers, including medical advice and lack of time. Higher levels of PA tended to be associated with better physical health status, supporting individualized, risk-based counseling and supervised strategies to safely promote PA in patients with arrhythmias.

## Introduction

Arrhythmias encompass a broad spectrum of cardiac rhythm disorders associated with increased morbidity and a substantially elevated risk of sudden cardiac death. Beyond their electrophysiological complexity, these conditions often generate uncertainty among patients and clinicians regarding the safety of engaging in physical activity (PA). Some individuals may exercise without adequate preparation or medical supervision, whereas others avoid PA altogether due to fear of triggering arrhythmic events. Both conditions may compromise health outcomes and quality of life (QoL).^1-4^

International recommendations for adults typically target 150-300 minutes per week of moderate-intensity aerobic activity (or 75-150 minutes per week of vigorous activity), along with muscle-strengthening activities on at least 2 days per week. Despite the broad recognition that regular PA is beneficial and generally safe for most cardiac patients, and the availability of evidence-based guidance on exercise prescription and supervision, real-world implementation remains limited. This gap is often driven by persistent uncertainty among both clinicians and patients.^1-4^

Several factors may contribute to this scenario, including challenges in risk stratification and individualized exercise prescription, limited familiarity with arrhythmia-specific considerations, and suboptimal recognition of the heterogeneity and clinical needs of patients with complex or inherited arrhythmias. These limitations may in turn contribute to lower PA levels in higher-risk subgroups, particularly when counseling is overly restrictive, inconsistent, or not tailored to the individual's arrhythmia phenotype and perceived triggers. In this context, precautionary advice and fear-avoidance behaviors may inadvertently promote sedentary patterns, even among patients with preserved LVEF.^1-4^

Identifying subgroups of patients at higher risk of physical inactivity or unsafe exercise practices is therefore essential to inform tailored recommendations and promote safe engagement in PA. Accordingly, this study aimed to describe PA levels and perceived barriers, and to explore their associations with arrhythmia phenotype and QoL in a tertiary outpatient cohort with documented arrhythmias and preserved LVEF, including inherited arrhythmia syndromes. By characterizing activity profiles and associated clinical factors, this study aims to identify patient groups that may benefit from enhanced counseling, structured exercise guidance, or targeted interventions to optimize safety and participation in PA.

## Methods

### Study design and setting

This was a prospective, cross-sectional clinical study conducted using a convenience sample of consecutive patients. As a feasibility-based pilot study, no formal a priori sample size calculation was performed; the target sample was defined by the expected recruitment capacity during the study period. Recruitment commenced following approval by the institutional Research Ethics Committee and the signing of informed consent forms.

With a sample size of n = 202, the precision around a 20% prevalence of physical inactivity is approximately ± 5.5% (95%CI), which is adequate for descriptive and hypothesis-generating comparisons across arrhythmia phenotypes in this pilot setting. However, confidence intervals widen in smaller phenotype subgroups.

### Study population

Eligible participants were consecutive adult outpatients with documented cardiac arrhythmias, including supraventricular tachycardia (SVT, including atrioventricular nodal reentrant tachycardia and atrioventricular reentrant tachycardia), atrial fibrillation (AF) and/or atrial tachycardia (AT) (including atrial flutter when applicable), ventricular ectopy/non-sustained ventricular tachycardia/ventricular tachycardia, inherited arrhythmia syndromes (e.g., long QT syndrome [LQTS] and Brugada syndrome), and/or prior cardiac arrest, followed at a tertiary arrhythmia clinic. All participants had preserved left ventricular ejection fraction (LVEF ≥ 50%) on transthoracic echocardiography.

Diagnoses were established through review of clinical records and documentation from 12-lead electrocardiography (ECG), Holter monitoring, exercise testing, and/or device interrogation. Inherited arrhythmia syndromes were identified based on accepted clinical criteria, including phenotype-specific ECG features, trigger profiles, and family history, with confirmatory provocative and/or genetic testing when available. Conditions such as Wolff-Parkinson-White syndrome and other inherited cardiac disorders were also diagnosed using ECG, Holter monitoring, or exercise testing.

Exclusion criteria included bedridden individuals, those with impaired ambulation or severe mobility limitations, advanced disease with life expectancy < 1 year, and enrollment in concurrent interventional studies. As recruitment was feasibility-based and consecutive, the number of screened but not enrolled patients was not prospectively recorded.

#### International Physical Activity Questionnaire

The International Physical Activity Questionnaire (IPAQ) is a standardized instrument used to assess PA levels in adults based on frequency, duration, and intensity. Participants reported activities performed over the previous 7 days, categorized as vigorous, moderate, or walking.

Participants were classified according to standard IPAQ categories: very active, active, irregularly active A, irregularly active B, and sedentary. The irregularly active A and B categories represent individuals who engaged in some PA but did not meet the frequency and/or duration criteria required for the active or very active categories. These were therefore treated as intermediate ("insufficiently active") categories rather than being merged with either active or sedentary groups.

For inferential analyses, the very active and active categories were combined due to the small number of very active participants (n = 7). Multinomial models compared four categories (active/very active, irregularly active A, irregularly active B, and sedentary), using sedentary as the reference category.^5^

### Assessment of quality of life

The 12-Item Short Form Survey (SF-12) is a validated, self-administered instrument that measures health-related QoL through two summary scores: the Physical Component Summary (PCS) and the Mental Component Summary (MCS), both ranging from 0 to 100, with higher scores indicating better perceived health.^6,7^

### Outcomes

The primary outcomes were i) the prevalence of physical inactivity as assessed by IPAQ; and ii) SF-12 PCS and MCS scores. Secondary outcomes included self-reported barriers to exercise and exploratory associations between PA status and QoL.

### Statistical analysis

Continuous variables are presented as mean ± standard deviation (SD) when approximately normally distributed, and as median (interquartile range) otherwise. Categorical variables are presented as counts and percentages (n [%]). Distributional assumptions were assessed visually using histograms and Q-Q plots.

Between-group comparisons for categorical variables were performed using Pearson's chi-square test or Fisher's exact test, as appropriate. Age across IPAQ categories was compared using one-way analysis of variance, with residual diagnostics performed to assess model assumptions.

The association between IPAQ category (active/very active, irregularly active A, irregularly active B, sedentary) and arrhythmia group was assessed using Pearson's chi-square test and further examined using multinomial logistic regression (reference outcome: sedentary; reference arrhythmia group: inherited), adjusted for age.

Associations between SF-12 PCS scores and IPAQ category were evaluated using multivariable linear regression models that were adjusted for age, sex, and symptom status. Models for MCS scores were adjusted for sex and symptom status only. A two-sided alpha of 0.05 was considered statistically significant. Due to the exploratory, hypothesis-generating nature of the study, no formal adjustment for multiple comparisons was applied.

## Results

### Sample demographic and clinical characteristics

The study population had a mean age of 50.5 ± 15.3 years, and 119 participants (58.9%) were male. Baseline demographic and clinical characteristics are summarized in Table 1. Most participants were asymptomatic (110 [54.4%]), and a minority had device therapy, including implantable cardioverter-defibrillators (ICDs) in 45 (22.3%) and pacemakers in 6 (3.0%).

**Table 1:** Baseline demographic and clinical characteristics

Variable		Value
Sample size, n		202
Age, years (mean ± SD)		50.5 ± 15.3
Male sex, n (%)		119 (58.9)
Hypertension, n (%)		65 (32.2)
Diabetes mellitus, n (%)		34 (16.8)
Asymptomatic status, n (%)		110 (54.4)
Implantable cardioverter-defibrillator, n (%)		45 (22.3)
Pacemaker, n (%)		6 (3.0)
BMI category, n (%)		
	Normal weight	62 (30.7)
	Overweight	81 (40.1)
	Obesity class I	39 (19.3)
	Obesity class II	9 (4.5)
	Obesity class III	10 (4.9)
	Missing values	1 (0.5)

BMI: body mass index; SD: standard deviation.

Arrhythmias were categorized into five groups: i) SVT, ii) ventricular arrhythmias, iii) inherited arrhythmias, iv) multiple arrhythmias, and v) AF and/or AT. Atrial flutter could be present in this cohort and is described among the documented arrhythmias; however, for predefined subgroup analyses, the atrial category was labeled as AF/AT to ensure consistency across tables and models. The baseline distribution of arrhythmia groups is presented in Table 2.

**Table 2:** Arrhythmia groups (pre-specified clinical classification)

Arrhythmia group	n (%)
SVT	26 (12.9%)
AF/AT	50 (24.8%)
Ventricular arrhythmias	81 (40.1%)
Inherited arrhythmias	37 (18.3%)
Multiple-arrhythmia	8 (4.0%)

AF: atrial fibrillation; AT: atrial tachycardia; SVT: supraventricular tachycardia.

### Physical activity

Overall IPAQ categories are presented in Table 3. In total, 41 participants (20.3%) were sedentary, 85 (42.1%) were active, and 7 (3.5%) were very active (combined active/very active: 92 [45.6%]). The prevalence of sedentary behavior was higher among participants with ventricular arrhythmias (25.9%), inherited arrhythmias (35.1%), and multiple arrhythmias (25.0%), compared with those with SVT (3.8%) and AF/AT (including AFL when applicable) (8.0%) (p = 0.043) (Figure 1; Table 4). These findings were observed despite preserved LVEF in all participants and a predominantly asymptomatic cohort (Table 1).

**Table 3:** Physical activity categories according to the IPAQ (short form)

IPAQ category	n (%)
Very active	7 (3.5%)
Active	85 (42.1%)
Irregularly active A	35 (17.3%)
Irregularly active B	34 (16.8%)
Sedentary	41 (20.3%)

IPAQ: International Physical Activity Questionnaire.

**Figure 1:** Proportion of sedentary participants according to arrhythmia group (IPAQ short form). AF: atrial fibrillation; AT: atrial tachycardia; IPAQ: International Physical Activity Questionnaire; SVT: supraventricular tachycardia.

**Table 4:** IPAQ classification by arrhythmia group

Arrhythmia group	Total, n	Very active, n	Active, n	Irregularly active A, n	Irregularly active B, n	Sedentary, n (%)
SVT	26	4	9	7	5	1 (3.8)
AF/AT	50	3	26	10	7	4 (8.0)
Ventricular arrhythmias	81	0	34	9	17	21 (25.9)
Inherited arrhythmias	37	0	13	8	3	13 (35.1)
Multiple arrhythmias	8	0	3	1	2	2 (25.0)

AF: atrial fibrillation; AT: atrial tachycardia; SVT: supraventricular tachycardia; IPAQ: International Physical Activity Questionnaire.

### Multinomial model for International Physical Activity Questionnaire classification

In multinomial logistic regression analysis (reference category: sedentary IPAQ; reference arrhythmia group: inherited; adjusted for age), the SVT and AF/AT groups had higher odds of being classified as active or very active, compared with the inherited arrhythmia group (Table 5).

**Table 5:** Multinomial logistic regression: active/very active vs sedentary (reference arrhythmia group: inherited; adjusted for age)

Predictor	OR	95%CI	p-value
Intercept	—	—	0.459
Age	1.01	0.99-1.04	0.308
SVT	12.06	1.36-106.90	0.025
AF/AT	5.77	1.52-21.96	0.010
Ventricular arrhythmias	1.42	0.54-3.74	0.479
Multiple arrhythmias	1.27	0.18-9.10	0.811

AF: atrial fibrillation; AT: atrial tachycardia; OR: odds ratio; SVT: supraventricular tachycardia.

### Quality of life

Most participants rated their health as good (65%) and reported minimal difficulty in daily activities. However, 39% showed lower physical performance, and 43% reported decreased mental performance. Pain and emotional distress were generally mild or absent. Men and asymptomatic individuals had higher PCS and MCS scores.

In adjusted models, active or very active PA showed a trend toward higher PCS scores (Table 6), whereas PA category was not independently associated with MCS scores (Table 7).

**Table 6:** Multivariable linear regression for SF-12 PCS score according to physical activity status, adjusted for age, sex, and symptom status

Predictor	β coefficient	SE	p-value
Intercept	47.38	2.55	< 0.001
Active/very active	2.94	1.67	0.080
Irregularly active A	0.23	2.08	0.910
Irregularly active B	–0.04	2.04	0.982
Age (per year)	–0.13	0.04	0.001
Male sex	1.70	1.29	0.189
*Asymptomatic status*	5.62	1.30	< 0.001

PCS: Physical Component Summary; SE: standard error; SF-12: 12-Item Short Form Survey.

**Table 7:** Multivariable linear regression for SF-12 MCS score according to physical activity status, adjusted for sex and symptom status

Predictor	β coefficient	SE	p-value
Intercept	42.83	1.82	< 0.001
Active/very active	–0.69	1.81	0.703
Irregularly active A	–0.69	2.23	0.757
Irregularly active B	0.82	2.22	0.711
Male sex	3.58	1.40	0.011
*Asymptomatic status*	3.48	1.40	0.014

MCS: Mental Component Summary; SE: standard error; SF-12: 12-Item Short Form Survey.

### Additional findings

The main reported reasons for physical inactivity were "other" (41.5%), lack of time (24.4%), and medical advice (19.5%). Despite this, most sedentary participants expressed a desire to engage in exercise. Following arrhythmia diagnosis, 66% of participants continued exercising, and 85% maintained sexual activity.

### Relationship between physical activity and quality of life

Participants classified as physically active had higher PCS scores compared with inactive individuals. Additionally, the absence of symptoms was associated with better QoL outcomes.

## Discussion

Research consistently demonstrates that higher levels of PA are associated with improved physical and psychological well-being. Current recommendations advise 150-300 minutes per week of moderate-intensity aerobic activity (or 75-150 minutes per week of vigorous activity), along with muscle-strengthening activities on at least 2 days per week. In patients with arrhythmias, exercise counseling should incorporate arrhythmia substrate, symptom burden, triggers, and device therapy. More vigorous or competitive exercise should be considered within an individualized framework based on risk stratification, optimization of therapy, and shared decision-making.^1-4^

Existing evidence has largely focused on phenotype-specific cohorts or device-based populations. In AF, the CHAMPLAIN-AF study (n = 619) reported a median of 100 minutes/week of moderate-to-vigorous PA (MVPA) and a sitting time of 6 hours/day. Notably, 56% of participants did not meet guideline-recommended targets, 54% were unaware or uncertain about MVPA recommendations, and 72% believed that PA should be part of AF management. Importantly, this cohort was not restricted to preserved LVEF and included patients with heart failure (HF) or cardiomyopathy. Similarly, device-based accelerometer studies (e.g., populations using implantable cardioverter-defibrillator or undergoing cardiac resynchronization therapy) have shown that lower PA levels are associated with HF hospitalization and mortality; however, these cohorts are typically enriched for decreased LVEF.

Accordingly, data specifically addressing arrhythmia outpatients with preserved LVEF remain limited. This raises the possibility that the diagnosis itself, along with overly restrictive or non-individualized counseling, may inadvertently promote sedentary behavior. Few studies have examined activity levels and modifiable barriers across distinct arrhythmia phenotypes within a single tertiary outpatient cohort with preserved LVEF.^8,9^ The novelty of the present study lies not in the direction of associations, but in the phenotype-stratified distribution of inactivity and modifiable barriers within this specific clinical context.

In this cohort, physical inactivity was common and clustered among clinically higher-risk phenotypes, including ventricular and inherited arrhythmia groups, although these findings should be interpreted cautiously due to smaller subgroup sizes (Figure 1). Sedentary prevalence was highest in the inherited arrhythmia group (35.1%) and remained elevated in the ventricular (25.9%) and multiple-arrhythmia (25.0%) groups, compared with SVT (3.8%) and AF/AT (8.0%). Notably, 45.6% of participants were classified as active or very active, and most (54.4%) were asymptomatic, reporting good overall health and minimal limitations in daily activities.

These findings suggest that factors beyond symptom burden, such as risk perception, counseling practices, medication effects, and practical constraints, may play a substantial role in limiting PA among higher-risk phenotypes. Among sedentary participants, the most frequently reported barriers were "other," lack of time, and medical advice to avoid exercise. Despite this, most expressed willingness to engage in PA (Figure 2).

**Figure 2:** Main reported barriers to physical activity among sedentary participants (n = 41). Bars represent the number and percentage of participants reporting each barrier. Notably, 39 of 41 participants (95.1%) expressed willingness to become physically active.

In age-adjusted multinomial models, SVT and AF/AT were associated with higher odds of being classified as active or very active, compared with inherited arrhythmias (Table 5). This supports a phenotype-specific pattern in which inherited arrhythmia substrates may be associated with greater restriction, heightened risk perception, or fear-avoidance behaviors, even in a predominantly asymptomatic cohort with preserved LVEF.

Beyond phenotype differences, PA level tended to be associated with health-related QoL in the physical domain. In adjusted linear models, active or very active individuals showed a trend toward higher SF-12 PCS scores compared with sedentary participants, whereas irregularly active categories did not differ substantially from sedentary individuals (Table 6). In contrast, SF-12 MCS scores were not clearly associated with activity categories and appeared to be more strongly influenced by symptom status and sex (Table 7).

Among sedentary participants, the leading reported barriers were lack of time and medical advice to refrain from exercise; however, most expressed willingness to become physically active. Together, these findings highlight a pragmatic implementation gap between guideline-based exercise recommendations and real-world counseling in tertiary arrhythmia care.

Baseline symptom status was not clearly associated with IPAQ categories, whereas it was consistently associated with SF-12 domains. This suggests that sedentary behavior may reflect not only symptom burden but also factors such as risk perception, counseling practices, and fear of triggering arrhythmias.

Despite broad recognition that regular PA is beneficial and generally safe for most cardiac patients, its implementation remains limited. This gap may be driven by challenges in risk stratification and individualized exercise prescription, limited familiarity with arrhythmia-specific considerations, and incomplete recognition of the heterogeneity and clinical needs of patients with complex or inherited arrhythmias.

Although this cohort was not designed to represent athletes or competitive sports participants, sports cardiology and disease-specific statements provide a practical, phenotype-based framework to contextualize exercise counseling in routine clinical care. Accordingly, guideline-oriented considerations by arrhythmia category are summarized in Table 8; these should not be interpreted as formal eligibility criteria for competitive sports participation.

**Table 8:** Guideline-oriented exercise recommendations and considerations by arrhythmia category (summary)

Arrhythmia category	General recommendation (summary)	Key references
SVT/pre-excitation (stable, controlled)	Recreational moderate-intensity aerobic activity is generally encouraged; individualized assessment is recommended in the presence of pre-excitation with high-risk features.	Pelliccia et al., 2021^2^
AF/AT	Regular physical activity is encouraged; target at least 150 min/week of moderate-intensity activity (or equivalent), when clinically appropriate. Shared decision-making should consider symptom burden, rate control, and comorbidities.	Pelliccia et al., 2021^2^; Joglar et al., 2024^4^
Ventricular arrhythmias/VT (preserved LVEF)	Exercise prescription should be individualized. Strenuous or competitive exercise should be avoided in higher-risk phenotypes until appropriate risk stratification and optimization of therapy; supervised programs may be appropriate.	Pelliccia et al., 2021^2^; Zeppenfeld et al., 2022^3^
Inherited arrhythmia syndromes (e.g., LQTS, Brugada syndrome, CPVT)	Exercise restrictions should be individualized according to genotype, phenotype, and trigger profile. Emphasis should be placed on physician-guided exercise planning and risk mitigation strategies.	Pelliccia et al., 2021^2^; Fernandez Gil et al., 2025^10^; Chen et al., 2022^11^
ICD recipients	Participation in competitive or vigorous sports was historically restricted; contemporary evidence supports individualized decision-making. Device programming, shared decision-making, and sport-specific risk assessment are essential.	Sharma et al., 2021^2^

AF: atrial fibrillation; AT: atrial tachycardia; CPVT: catecholaminergic polymorphic ventricular tachycardia; ICD: implantable cardioverter-defibrillator; LVEF: left ventricular ejection fraction; LQTS: long QT syndrome; SVT: supraventricular tachycardia; VT: ventricular tachycardia.

Inherited arrhythmia syndromes often occur in the absence of overt structural heart disease. Available evidence suggests high rates of sedentary behavior in certain populations (e.g., Brugada syndrome cohorts), supporting the hypothesis that diagnosis-related concerns, counseling practices, and fear-avoidance behaviors may contribute to lower PA levels, even when systolic function is preserved. Conversely, in LQTS, some cohorts report self-reported activity levels comparable to controls but decreased exercise performance, highlighting the potential influence of treatment (e.g., beta-blockers) and perceived limitations.^10,11^

Pharmacotherapy may also influence exercise behavior and tolerance. Beta-blockers, a cornerstone therapy across multiple arrhythmia contexts, may blunt chronotropic response and reduce perceived exercise capacity, potentially reinforcing inactivity.^12,13^ This underscores the importance of individualized counseling to distinguish expected pharmacological effects from true exercise intolerance as well as the role of supervised exercise programs in facilitating safe and progressive activity while maintaining arrhythmia control.^2-4^

Overall, these findings support the systematic assessment of PA and its barriers using standardized tools, followed by risk-based counseling. They also highlight the need for structured care pathways, potentially integrated within cardiac rehabilitation programs, to deliver supervised exercise prescriptions, particularly for higher-risk phenotypes. The main contribution of this study is to provide pragmatic, real-world data from a tertiary arrhythmia outpatient cohort with preserved LVEF, including the distribution of inactivity across clinically relevant subgroups and the identification of modifiable barriers.

### Limitations of study

PA was self-reported using the IPAQ, which may be subject to recall bias, and the cross-sectional design precludes causal inference. This was a single-center tertiary referral cohort with preserved LVEF; therefore, the findings may not be generalizable to community-based arrhythmia populations or to patients with decreased LVEF. In addition, subgroup estimates were imprecise for smaller phenotypes, particularly the multiple-arrhythmia group. We did not assess participation in competitive sports, objectively measured PA, or detailed pharmacotherapy dosing and intensity.

## Conclusion

In a tertiary outpatient cohort of patients with arrhythmias and preserved LVEF, physical inactivity clustered among higher-risk phenotypes and was frequently associated with potentially modifiable barriers, including medical advice and lack of time. Higher levels of PA tended to be associated with better physical health status, supporting the need for individualized, risk-based counseling and supervised pathways to safely promote PA in arrhythmia populations.

## Data Availability

The underlying content of the research text is contained within the manuscript.
